# A Comprehensive and Person-Centered View of the Association Between the Dark Triad and Youth Mental Health

**DOI:** 10.3389/fpsyt.2022.900354

**Published:** 2022-06-30

**Authors:** Yunjun Hu, Xiaoyu Lan

**Affiliations:** ^1^Department of Students’ Affairs, Wenzhou University of Technology, Wenzhou, China; ^2^Promenta Research Center, Department of Psychology, University of Oslo, Oslo, Norway

**Keywords:** mental health, dark triad, high school students, person-centered approach, personality

## Abstract

Using a dual person-centered approach, the current study examined the Dark Triad profiles and mental health profiles among a large-scale sample of high school students. The study also simultaneously examined whether the emerging Dark Triad profiles could diverge in mental health profiles, delineating a thorough, and person-centered view of this association. To achieve these research aims, 1,640 Chinese high school students (*M*_age_ = 16.78; *SD* = 0.68; 57.6% females) participated in this study, and they were uniformly instructed to complete a set of well-established questionnaires. Results from latent profile analyses revealed five Dark Triad profiles—low Machiavellianism-psychopathy (7.4%), benevolent (61.7%), highly malevolent (6.7%), low narcissism (8.8%), and malevolent (15.4%)—and the following four mental health profiles: flourishing (37.7%), vulnerable (16.4%), troubled (33.9%), and highly troubled (12.4%). Moreover, results from multiple multinomial regression analyses showed that, among all five empirically derived Dark Triad profiles, students with the low Machiavellianism-psychopathy profile exhibited the highest probability of being “flourishing,” whereas those with the low narcissism profile showed the highest likelihood of being “highly troubled.”

## Introduction

During high school, students are increasingly exposed to a wide range of challenging circumstances and academic-related demands. For instance, students expect increased autonomy from their parents and should gradually navigate ever-more complicated peer dynamics (1, 2). Students in high school settings also often experience salient academic-related stress due to ongoing academic demands, which may precipitate the risk of mental illness (3). In accordance with these concerns, empirical studies have confirmed that psychological distress during this period of life could have long-term detrimental impacts on both physical and mental health later in adulthood (4, 5). Investigating the correlates of high school students’ mental health can therefore provide novel insights into evidence-based intervention or prevention programs to facilitate personal growth.

Nevertheless, the assumption that the population is homogeneous has often resulted in intervention or prevention programs being administered to youth populations without elaborative consideration of personal characteristics that differentiate the intervention processes. In the current study, we extended prior research by using a dual person-centered approach to investigate high school students’ mental health profiles and the Dark Triad profiles; our aim was to examine the associations between the profiles, delineating a thorough and person-centered view of the associations. Extending the literature in this field is not only theoretically relevant to a dual-factor model of mental health and the Dark Triad structure, but also practically informative when precisely locating vulnerable or at-risk students to potentially improve the efficiency of targeted intervention or prevention programs.

In what follows, we conduct a literature review concerning each study component (i.e., mental health and Dark Triad) and the associations between the components, starting with a review of research on youth mental health.

### Youth Mental Health

Mental health is traditionally defined by the absence of psychopathology and associated levels of impaired functioning (6, 7). However, in recent decades, this narrow view has been challenged by the rising expansion of positive psychology (8) and increasing recognition that the absence of mental illness is not identical to the presence of positive mental health (9, 10). Distinct from this traditional view, a dual-factor model of mental health posits that individuals’ mental health should be regarded as a complete state of being, one in which negative (i.e., psychopathology) and positive (i.e., subjective wellbeing) mental health indicators are not extremes on a single continuum (11). Psychopathology encompasses both internalizing problems (e.g., depression and anxiety) and externalizing problems (e.g., aggressive behavior and rule-breaking behavior) (12, 13). Subjective wellbeing, on the other hand, entails three related yet separate constructs: life satisfaction (cognitive appraisal of one’s quality of life), positive affect (positive emotional experiences), and negative affect (negative emotional experiences) (14). Based on different combinations of psychopathology and subjective wellbeing, four mental health groups can be derived: (a) troubled (high psychopathology and low wellbeing), (b) flourishing (low psychopathology and high wellbeing), (c) symptomatic but content (high psychopathology and high wellbeing), and (d) vulnerable (low psychopathology and low wellbeing).

Many empirical studies have indicated the importance of considering psychopathology and subjective wellbeing in tandem, which aligns with a dual-factor model of mental health (15, 16). These studies indicate that, for sizable groups of individuals, psychopathology scores co-occur with subjective wellbeing values. Given this co-occurrence, researchers should simultaneously incorporate psychopathology and subjective wellbeing when investigating the correlates of youth mental health. Nevertheless, empirical studies relying on a dual-factor model of mental health have mostly employed predetermined cutoff scores to identify group membership. Such an approach relies on an arbitrarily assigned distance between groups and does not require additional external discriminant analyses to examine how different groups relate to indicators (17). To gain a more comprehensive understanding of youth mental health, we aimed to apply a person-centered approach that can unravel relatively homogeneous groups of adolescents with distinct configurations in terms of psychopathology and subjective wellbeing.

Further, although numerous studies on high school students’ mental health have been conducted, insufficient research has focused on the mental health of Chinese high school students, who often encounter considerable academic pressure deriving from the high-stakes National College Entrance Examination (18).

### Study Context in China

Chinese students, especially high school ones, are generally overburdened by the pressures of maintaining high levels of academic excellence. Such pressures are the result of academic competence in East Asian societies being more strongly linked to family dignity, high social status, occupational competitiveness, and job wages than in many individualistic societies, such as North America (3, 19). Due to these cultural and societal emphases, Chinese parents tend to have extremely high expectations for children’s academic performance (3, 19). Chinese students often, due to those same emphases, respect their parents’ authority and are less likely to openly contradict parental expectations (20, 21). Under high parental expectations and salient academic pressure, Chinese students indeed show superior academic performance through diligence and perseverance but also exhibit higher levels of anxiety and emotional distress than their peers from individualistic societies (22).

In individualistic societies, students often view themselves as independent from others and focus on individual volition, making the students more likely to seek social support under academic and social pressures with little caution. The underlying cultural assumptions lead such students to believe they can proactively foster their own wellbeing and that others, based on their own willingness, are also free to assist (23, 24). By contrast, in Chinese society, individuals are socialized to hold a highly interdependent self-construal. Chinese individuals therefore tend to be highly sensitive to social cues and potentially more likely to feel social anxiety and emotional distress than individuals from individualistic societies, for instance, who often demonstrate a high independent self-construal (25, 26). Moreover, Chinese society heavily emphasizes establishing and maintaining the harmony of the social group. Given these prominent features of Chinese society, students are reluctant to explicitly ask for support from close others when encountering emotional distress and stressful life events; bringing personal difficulties to the attention of others within the social group can erode the harmony and improperly demand individuality (23, 27, 28). Additionally, Chinese students are less likely than their peers from individualistic societies to seek professional counseling services when encountering psychological distress; visiting a psychologist is highly stigmatized and considered a threat to the dignity of the whole family (29). Substantial empirical studies have documented that Chinese high school students exhibit a high prevalence of emotional distress (3–5, 18). Given the described cultural emphases, this line of research can be extended by focusing on the mental health of Chinese high school students.

In addition, to highlight intervention efforts in schools, the focus should be on the relatively stable traits, such as personality, that fundamentally impact youth mental health (30). Regarding personality traits, we focused on the Dark Triad in the current study, as discussed below.

### Dark Triad

Dark Triad refers to the constellation of three conceptually distinct but empirically overlapping personality traits: Machiavellianism, narcissism, and psychopathy (31). Specifically, Machiavellianism refers to those who lack empathy toward others and tend to be deceptive and manipulative; narcissism involves those who are characterized by a grandiose self-view and an increased sense of entitlement; and psychopathy indicates those who are impulsive, self-centered, and emotionally cold and who tend to exhibit antisocial behaviors (31–33). Prior research has examined the role of benevolent and neutral personality traits in youth mental health within mainstream personality structures, such as the Big Five. Comparatively, fewer studies have focused on malevolent personality factors, such as the Dark Triad, in youth mental health (34). Prior research has, however, demonstrated that the Dark Triad incrementally predicts psychological outcomes above and beyond the Big Five (31, 35–37). This incremental value merits further research on Chinese youth populations, complementing the extant literature on the Dark Triad and enriching the disproportionate amount of the literature focused on the link between the Dark Triad and positive/negative psychological outcomes. In addition, examining the association between the Dark Triad and youth mental health is essential because high school students confront salient socioemotional changes and academic pressures, which could amplify malevolent traits (34). Götz et al. for instance, demonstrated that Machiavellianism peaked around age 16 (34). In this perspective, studying the Dark Triad in the context of high school students provides a critical contribution to the literature due to salient manifestations of socially aversive traits during this period of life.

To date, extensive studies have relied on a variable-centered approach to study the Dark Triad traits. This approach is informative and valuable, but ultimately fails to represent a broad spectrum of personalities based on their natural configurations. For instance, individuals may simultaneously possess more than one Dark Triad trait (36). Given this limitation, we aimed to utilize a person-centered approach to identify relatively homogeneous groups of youth with distinct configurations in terms of Machiavellianism, narcissism, and psychopathy. Applying such an approach regards the individual as a whole system and allows for exploring how the Dark Triad traits operate conjointly and within youth (38–40).

Among extant but limited literature on applying a person-centered approach to the Dark Triad, Nguyen et al. (38) found four Dark Triad profiles among a sample of French adults: benevolent (low scores on all three dimensions), high Machiavellianism (high scores on Machiavellianism but low scores on narcissism and psychopathy), high psychopathy (high scores on psychopathy but low scores on Machiavellianism and narcissism), and malevolent (high scores on all three dimensions) (38). Chabrol et al. (41) also reported four profiles based on the Dark Tetrad traits (the Dark Triad plus sadism) in French high school students: low-traits, sadistic-Machiavellian, psychopathic-narcissistic, and high-traits (41). In addition, Kam and Zhou (42) identified three Dark Triad profiles among Canadian undergraduate students: low, intermediate, and high levels of each trait (42). The findings from Kam and Zhou notably indicate that the Dark Triad is unidimensional, and they argued against applying a person-centered approach to study the Dark Triad. Similar results have been recently replicated by Garcia and MacDonald (43) in a sizable Western-based sample (43). Despite these tremendous strides, however, the mounting but limited literature on the Dark Triad and Tetrad profiles still exhibits conflicting evidence that requires further investigation.^1^ Moreover, these empirical studies center on adults from Western societies, restricting the generalization of research findings. Considering the developmental nature of youth and the unique cultural features in which youth are embedded, the literature can be meaningfully extended by exploring the Dark Triad profiles in youth from Eastern societies, such as China.

### Association Between the Dark Triad and Youth Mental Health

In terms of the association between the Dark Triad and psychosocial indicators, a recent meta-analysis reported that Dark Triad traits are linked to adverse psychosocial indicators but not positive outcomes (30). More precisely, Machiavellianism, especially psychopathy, exhibits a stronger association with adverse psychosocial outcomes than narcissism. Muris et al. (30) found that psychopathy is the dominant malevolent trait, explaining unique variance in all psychosocial outcomes; by contrast, Machiavellianism and narcissism seemingly do not make independent contributions (30). In terms of Chinese adolescents, Li et al. (44) found that Machiavellianism and psychopathy are negatively linked to subjective wellbeing, while narcissism is positively related to subjective wellbeing (44). In terms of negative outcomes of mental health, Zhang et al. (45) showed that Machiavellianism and psychopathy are positively associated with loneliness, while narcissism is negatively related to loneliness (45). Similarly, findings from Geng et al. (46) and Geng et al. (47) also confirmed the negative association of Machiavellianism and psychopathy to internalizing and externalizing problems in Chinese adolescents (46, 47).

These findings notwithstanding, no empirical studies have investigated the Dark Triad profiles and then examined how differing Dark Triad profiles may simultaneously diverge in mental health profiles. Therefore, using a person-centered approach to identify the Dark Triad profiles and then investigate their associations with separated outcomes merely yields a limited view of this association.

### The Present Research

To address the limitations in the existing literature, the current study aimed to apply a dual latent profile analysis to probe both mental health profiles (indexed by psychopathology and subjective wellbeing) and Dark Triad profiles (indexed by Machiavellianism, narcissism, and psychopathy) in a large-scale sample of Chinese high school students. We therefore empirically identified these profiles and then examined whether differing Dark Triad profiles diverge in mental health profiles. Specifically, the current study examined the following research questions (RQ):

RQ1: How many meaningful mental health profiles among Chinese high school students can be identified?

RQ2: How many meaningful Dark Triad profiles among Chinese high school students can be derived?

RQ3: Do differing Dark Triad profiles diverge with respect to mental health profiles among Chinese high school students?

Based on the current literature review, we generated specific hypotheses for each RQ. For RQ1, we expected four mental health profiles: troubled, flourishing, symptomatic but content, and vulnerable. For RQ2, we did not formulate a specific hypothesis due to inconsistent findings identified, but, according to the extant research, we expected to derive three or four profiles characterizing differing degrees of the three socially aversive traits. Concerning RQ3, we hypothesized that students within the profile characterized by low Machiavellianism and psychopathy but high narcissism are more likely to be the flourishing profile, whereas students within the profile characterized by high Machiavellianism and psychopathy but low narcissism are more likely to be the troubled profile. Notably, we statistically controlled for age, gender, and family socioeconomic status when addressing all RQs because prior research has suggested that these sociodemographic variables could not only influence mental health outcomes and the Dark Triad, but also impact the strength of the associations between the two (48–50).

## Materials and Methods

### Participants and Procedures

Before data collection, ethics approval was obtained from the principal investigator’s affiliation. We first contacted school principals and head teachers in public high schools, and explained the objectives of this research project. After getting their approval, we invited students to participate in this investigation voluntarily. Data were collected with the help of trained graduate students and head teachers in each classroom during a regular class hour. Confidentiality, anonymity, and participants’ rights were strictly guaranteed during all research processes. The final sample comprised 1,640 Chinese high school students (57.6% female; *M*_age_ = 16.78; *SD* = 0.68). At the time of data collection, participants attended 10th (*n* = 918; 56%) and 11th (*n* = 722; 44%) grades in six public high schools in China.

### Measures

#### Internalizing and Externalizing Problems

Internalizing and externalizing problems were assessed by the Chinese adaptation of the Youth Self-Report (YSR) (12, 51). The Chinese version of the YSR consists of 30 items and two dimensions: internalizing problems (15 items; e.g., “I am too fearful or anxious”) and externalizing problems (15 items; e.g., “I destroy my own things”). Students were asked to assess each item based on a 4-point scale ranging from 1 (*definitely does not apply to me*) to 4 (*definitely applies to me*). Following previous research (51), the score was calculated by averaging all items separated by two dimensions. Higher scores indicated greater internalizing problems and externalizing problems, respectively. Previous studies have shown good internal consistency of the YSR in Chinese adolescents (29, 52). In this study, Cronbach’s alpha was 0.92 for internalizing problems and 0.89 for externalizing problems.

#### Subjective Wellbeing

Life satisfaction was assessed by the Multidimensional Students’ Life Satisfaction Scale (MSLSS) (53). The MSLSS contains 25 items and assesses five life domains (family, friends, school, living environment, and self). One of the item examples is, “I enjoy being at home with my family (on the family domain).” Participants were asked to rate each item on a 4-point Likert scale ranging from 1 (*totally disagree*) to 4 (*totally agree*). The average score of these items was calculated, with high values indicating higher levels of global life satisfaction. According to prior research (54), the MSLSS has demonstrated good internal consistency in Chinese adolescents. In this study, Cronbach’s alpha was 0.93.

Positive and negative affect were assessed using the 14-item Affect Balance Scale (ABS) developed by Bradburn (55). The ABS consists of two dimensions: positive affect (8 items; e.g., I am optimistic about the future) and negative affect (6 items; e.g., I feel so restless that I cannot sit long in a chair). Participants were asked to rate each item according to each experience’s frequency in their daily life on a 4-point scale ranging from 1 (*never*) to 4 (*always*). The average score for each dimension was calculated, with a higher score indicating a higher level of a particular affect. Based on previous studies on Chinese adolescents (49, 56), this scale has exhibited good internal consistency. In this study, Cronbach’s alpha was 0.85 for positive affect and 0.79 for negative affect.

#### Dark Triad

The Dark Triad was measured by the Short Dark Triad scale (SD3) (33), which has been validated in the Chinese cultural context, showing good psychometric properties (57). The SD3 consists of 28 items with 10 items for Machiavellianism, 9 items for narcissism, and 9 items for psychopathy. Item examples are, “most people can be manipulated (Machiavellianism); I like to get acquainted with important people (narcissism); I like to get revenge on authorities (psychopathy).” Each item was rated on a 5-point Likert scale ranging from 1 (*strongly disagree*) to 5 (*strongly agree*). Scores were established by averaging the values of all items, separated by each personality trait. A higher score indicated a greater tendency to each Dark Triad trait. Prior research has shown good internal consistency of the SD3 in Chinese youth (58). In this study, Cronbach’s alpha was 0.78 for Machiavellianism, 0.70 for narcissism, and 0.71 for psychopathy.

#### Sociodemographic Characteristics

Students were asked to report their age, gender, and family socioeconomic status. Family socioeconomic status was measured by parental educational level, parental occupational status, and family monthly income (58, 59). A composite standardized score was calculated, with high values indicating high levels of family socioeconomic status.

### Data Analysis

We used SPSS 28.0 and Mplus 7.0 to analyze the data (60, 61). Prior to addressing research questions, we conducted descriptive statistics (means and standard deviations) and Pearson’s correlations to have an overview of study variables. Little’s missing completely at random (MCAR) test was adopted to evaluate the influence of missing data (62). The MCAR showed that the missing data for all study variables were randomly distributed. In this context, the remaining missing values were computed using full information maximum likelihood (63).

In terms of RQ1 and RQ2, we applied latent profile analyses to identify mental health profiles and the Dark Triad profiles, after controlling for students’ age, gender, and family socioeconomic status. To determine the optimal number of latent profiles, we employed the following fit indices: Akaike information criteria (AIC), Bayesian information criterion (BIC), adjusted BIC (BIC), Lo-Mendell-Rubin adjusted likelihood ratio test (LMR-LRT), bootstrapped likelihood ratio test (BLRT), and entropy (39, 64). For AIC, BIC, and aBIC, lower values indicate a better model fit. For the LMR-LRT and BLRT, they compare models for *k* and *k*-1 profiles. If these values exhibit significant *p*-values, it suggests that the *k* profile model is a better fit than the *k*-1 profile model. With regard to entropy, higher scores indicate fewer classification errors, and scores higher than 0.70 usually suggest that the latent profiles highly discriminate. Apart from these fit indices, we also took the proportion of the smallest profiles into account. For example, solutions with small numbers of participants (e.g., less than 5% of the total sample) should be avoided, as they are not meaningful and lack practical value (65). To examine whether emerging latent profiles significantly differed on the selected indicators, multivariate analysis of variance (MANOVA) and the follow-up *post-hoc* test were conducted (40). Finally, z-scores of the observable indicators were calculated to aid the interpretation of the latent profile solution.

Regarding RQ3, we applied a multinomial logistic regression using maximum likelihood estimation to determine whether differing Dark Triad profiles may diverge in mental health profiles, after controlling for students’ age, gender, and family socioeconomic status.

## Results

### Descriptive Statistics and Correlations

Descriptive statistics and correlations for study variables are presented in Table 1. Machiavellianism and psychopathy were positively associated with negative indicators of mental health (i.e., internalizing problems, externalizing problems, and negative affect) and negatively linked to positive indicators of mental health (i.e., life satisfaction and positive affect); by contrast, narcissism was negatively related to these negative indicators (except for externalizing problems), and positively linked to positive indicators.

**TABLE 1:** Descriptive statistics and bivariate correlations of study variables for Chinese high school students.

	*M*	*SD*	Range	1	2	3	4	5	6	7	8	9	10	11
1. Machiavellianism	2.98	0.68	1–5	–										
2. Narcissism	2.85	0.65	1–5	0.28***	–									
3. Psychopathy	2.42	0.66	1–5	0.54***	0.38***	–								
4. Internalizing problems	1.80	0.60	1–4	0.20***	–0.12***	0.22***	–							
5. Externalizing problems	1.50	0.43	1–4	0.22***	0.01	0.40***	0.67***	–						
6. Life satisfaction	3.23	0.44	1–4	–0.13***	0.27***	–0.17***	–0.49***	–0.47***	–					
7. Positive affect	3.22	0.50	1–4	–0.13***	0.23***	–0.11***	–0.41***	–0.32***	0.56***	–				
8. Negative affect	2.41	0.59	1–4	0.15***	–0.08***	0.15***	0.62***	0.38***	–0.34***	–0.30***	–			
9. Age	16.78	0.68	16–18	0.02	–0.02	0.01	–0.01	0.03	–0.04	–0.01	–0.04	–		
10. Gender^a^	–	–	1–2	–0.19***	–0.11***	–0.20***	0.05*	–0.14***	0.08***	0.11***	0.08***	–0.03	–	
11. Socioeconomic status	0.00	3.75	–9.84–12.22	0.10***	0.10***	0.08***	–0.04	–0.01	0.04	–0.01	–0.03	–0.01	–0.03	–

N = 1,640. ^a^Coded as 1, male and 2, female. *p < 0.05, ***p < 0.001.

### Identifying Mental Health Profiles

Table 2 shows the goodness of fit indices for mental health profiles. As shown in Table 2, the likelihood ratio statistical tests were significant for the two-, three-, and four-profile solutions. Compared to the two- and three-profile solutions, the four-profile solution exhibited lower AIC, BIC, and aBIC, as well as an acceptable value of the entropy. Therefore, the four-profile solution was regarded as the optimal model in this study.

**TABLE 2:** The goodness of fit indices for mental health profiles.

	AIC	BIC	aBIC	Entropy	LMR- LRT	BLR	Smallest profiles (%)
1-Profile	12278.82	12332.84	12301.08	–	–	–	–
2-Profile	10195.03	10281.47	10230.64	0.82	2049.63***	2095.78***	38.2%
3-Profile	9625.48	9744.34	9674.45	0.82	568.74***	581.55***	13.4%
**4-Profile**	**9449.74**	**9601.01**	**9512.06**	**0.75**	183.60***	187.73***	**12.4%**
5-Profile	9328.73	9512.42	9404.41	0.73	130.07	133.00	10.7%

N = 1,640. The optimal model is highlighted in bold type. ***p < 0.001.

Further, Table 3 provides a summary of the means and standard deviations of each selected indicator across these four mental health profiles, as well as the *F*-values, partial eta-squared values, and *post hoc* comparisons of these profiles. The results of MANOVA and *post-hoc* tests showed statistically significant differences in all five indicators between profiles (see Table 3).

**TABLE 3:** Mean differences in study indicators across four mental health profiles.

	1. Flourishing (*n* = 611)		2. Vulnerable (*n* = 270)		3. Troubled (*n* = 556)		4. Highly troubled (*n* = 203)				
	*M*	*SD*	*M*	*SD*	*M*	*SD*	*M*	*SD*	*F*	Partial η ^2^	*Post hoc*
Internalizing problems	1.30	0.26	1.45	0.24	2.14	0.27	2.88	0.36	2079.33***	0.79	4 > 3 > 2 > 1
Externalizing problems	1.19	0.19	1.32	0.24	1.72	0.32	2.10	0.45	650.37***	0.54	4 > 3 > 2 > 1
Life satisfaction	3.61	0.27	2.98	0.28	3.14	0.33	2.69	0.37	574.41***	0.51	1 > 3 > 2 > 4
Positive affect	3.57	0.32	2.93	0.39	3.17	0.40	2.68	0.52	340.57***	0.38	1 > 3 > 2 > 4
Negative affect	2.03	0.49	2.29	0.44	2.63	0.44	3.07	0.49	317.80***	0.36	4 > 3 > 2 > 1

N = 1,640. ***p < 0.001.

**FIGURE 1:**
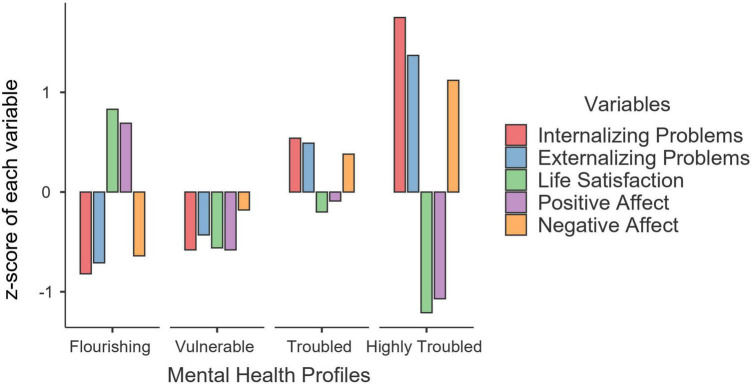
Four mental health profiles based on the z-value of each indicator. *N* = 1,640.

We referred to the dual-factor model of mental health (11) and z-scores of the observable indicators to interpret these profiles (see Figure 1). Specifically, students in the first profile (*n* = 611; 37.7%) reported the lowest scores on negative indicators (i.e., internalizing and externalizing problems, negative affect) and the highest scores on positive indicators (i.e., life satisfaction and positive affect); thus, this profile was labeled as “flourishing.” Students in the second profile (*n* = 270; 16.4%) scored low on all indicators; hence, this profile was marked as “vulnerable.” Students in the third profile (*n* = 556; 33.9%) reported high scores on negative indicators and low scores on positive indicators; therefore, this profile was named “troubled.” Students in the fourth profile (*n* = 203; 12.4%) were characterized by the highest scores on negative indicators and the lowest scores on positive indicators; therefore, this profile was named “highly troubled.”

### Identifying the Dark Triad Profiles

Table 4 presents the goodness of fit indices for the Dark Triad profiles. As shown in this table, the likelihood ratio statistical tests (i.e., LMR-LRT and BLRT) were significant for the two-, three-, and five-profile solutions. Compared to the two- and three-profile solutions, the five-profile solution exhibited lower AIC, BIC, and aBIC, as well as an acceptable value of the entropy. Given these results, the five-profile solution was regarded as the optimal model in this study.

**TABLE 4:** The goodness of fit indices for the Dark Triad profiles.

	AIC	BIC	aBIC	Entropy	LMR- LRT	BLR	Smallest profiles (%)
1-Profile	9980.68	10013.10	9994.03	–	–	–	–
2-Profile	9229.89	9283.92	9252.15	0.75	733.99***	758.78***	22.7%
3-Profile	9090.90	9166.53	9122.06	0.62	142.19***	146.99***	11.5%
4-Profile	9066.03	9163.27	9106.09	0.61	31.80	32.87	7.8%
**5-Profile**	**9032.34**	**9151.18**	**9081.30**	**0.70**	43.63*	45.11*	**6.6%**

N = 1,640. The optimal model is highlighted in bold type. *p < 0.05, ***p < 0.001.

Further, Table 5 provides a summary of the means and standard deviations of each indicator across these five Dark Triad profiles. MANOVA and *post-hoc* tests identified statistically significant differences between the profiles, except for no significant differences in narcissism between the second and fourth profiles, between the first and second profiles, and between the first and fourth profiles fourth profile.

**TABLE 5:** Mean differences in study indicators across five Dark Triad profiles.

	1. Low Machiavellianism-psychopathy (*n* = 121)		2. Benevolent (*n* = 1,012)		3. Highly malevolent (*n* = 109)		4. Low narcissism (*n* = 145)		5. Malevolent (*n* = 253)				
	*M*	*SD*	*M*	*SD*	*M*	*SD*	*M*	*SD*	*M*	*SD*	*F*	Partial η ^2^	*Post hoc*
Machiavellianism	1.79	0.29	2.76	0.37	4.20	0.35	3.97	0.29	3.33	0.33	1105.78***	0.73	3 > 4 > 5 > 2 > 1
Narcissism	2.56	0.55	2.67	0.51	3.86	0.62	2.54	0.47	3.45	0.51	238.54***	0.36	3 > 5 > 2 > 1 > 4
Psychopathy	1.59	0.39	2.20	0.44	3.61	0.47	2.61	0.49	3.06	0.43	498.32***	0.54	3 > 5 > 4 > 2 > 1

N = 1,640. ***p < 0.001.

**FIGURE 2:**
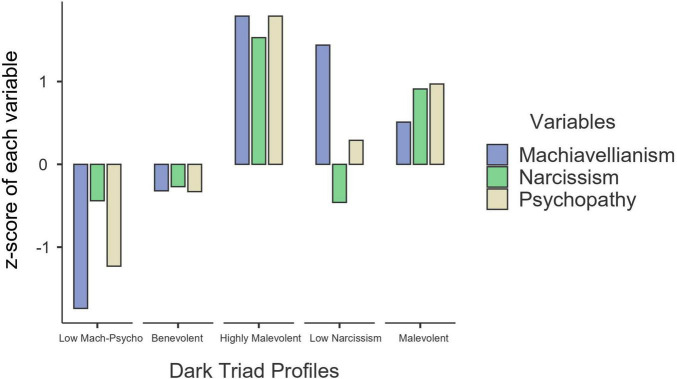
Five personality profiles based on the *z*-value of three Dark Triad traits. *N* = 1,640. Mach-Psycho = Machiavellianism-Psychopathy.

To interpret these profiles, we referred to prior research of the Dark Triad profiles (38) and z-scores of the observable indicators (see Figure 2). To be specific, students in the first profile (*n* = 121; 7.4%) reported the lowest scores on Machiavellianism and psychopathy and low scores on narcissism; thus, this profile was labeled “low Machiavellianism-psychopathy.” Students in the second profile (*n* = 1012; 61.7%) scored low on all dark personality traits; hence, this profile was marked “benevolent.” Students in the third profile (*n* = 109; 6.7%) reported the highest scores on all Dark Triad traits; therefore, this profile was named “highly malevolent.” Students in the fourth profile (*n* = 145; 8.8%) were characterized by high scores on Machiavellianism and psychopathy but low scores on narcissism; given this, this profile was named “low narcissism.” Students in the fifth profile (*n* = 253; 15.4%) reported high scores on all Dark Triad traits; hence, this profile was labeled “malevolent.”

### The Association of the Dark Triad Profiles With Mental Health Profiles

**FIGURE 3:**
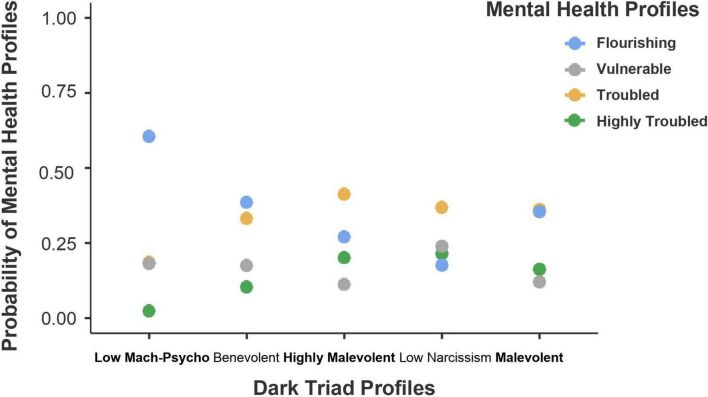
The effect of the Dark Triad profiles on the probability of mental health profiles. *N* = 1,640. Mach-Psycho, Machiavellianism-Psychopathy.

We applied a multinomial logistic regression to investigate their associations. In this step of the analysis, the flourishing profile was regarded as the reference group compared with the other three “maladaptive” profiles. The results of a multinomial logistic regression can be found in Table 6. To ease the interpretations of the results, we referred to a visualized graph (see Figure 3).

**TABLE 6:** Multiple multinomial regression analysis predicting mental health profiles.

Profiles contrast	Variables	*b*	*SE b*	95% CI for *b*		Odds Ratio	*t*	*p*
Vulnerable vs. flourishing	Benevolent vs. low Mach-Psycho	0.10	0.11	–0.11	0.31	1.11	0.93	0.35
	**Highly malevolent vs. Low Mach-Psycho**	–**0.36**	**0.15**	–**0.65**	–**0.06**	**0.70**	–**2.36**	**0.02**
	Low narcissism vs. Low Mach-Psycho	–0.02	0.02	–0.06	0.02	0.98	–0.98	0.33
	Malevolent vs. Low Mach-Psycho	0.41	0.27	–0.12	0.94	1.51	1.51	0.13
	Highly malevolent vs. Benevolent	0.32	0.42	–0.51	1.15	1.37	0.75	0.45
	**Low narcissism vs. Benevolent**	**1.50**	**0.37**	**0.79**	**2.22**	**4.49**	**4.11**	**<0.001**
	Malevolent vs. Benevolent	0.12	0.33	–0.53	0.77	1.13	0.36	0.72
	Low narcissism vs. Highly Malevolent	–0.09	0.35	–0.77	0.59	0.91	–0.27	0.79
	**Malevolent vs. highly Malevolent**	**1.09**	**0.28**	**0.55**	**1.64**	**2.98**	**3.95**	**<0.001**
	Malevolent vs. low Narcissism	–0.29	0.23	–0.74	0.16	0.75	–1.27	0.21
	**Age**	**1.19**	**0.42**	**0.36**	**2.01**	**3.27**	**2.81**	**0.01**
	Gender^a^	–0.20	0.39	–0.97	0.57	0.82	–0.51	0.61
	**Socioeconomic status**	–**1.38**	**0.33**	–**2.04**	–**0.73**	**0.25**	–**4.16**	**<0.001**
Troubled vs. flourishing	Benevolent vs. Low Mach-Psycho	–0.02	0.09	–0.19	0.15	0.98	–0.23	0.82
	Highly malevolent vs. Low Mach-Psycho	0.13	0.12	–0.11	0.38	1.14	1.08	0.28
	**Low narcissism vs. Low Mach-Psycho**	–**0.05**	**0.02**	–**0.08**	–**0.02**	**0.95**	–**3.06**	**0.01**
	**Malevolent vs. low Mach-Psycho**	**1.03**	**0.25**	**0.54**	**1.52**	**2.80**	**4.16**	**<0.001**
	**Highly malevolent vs. Benevolent**	**1.60**	**0.34**	**0.94**	**2.27**	**4.97**	**4.72**	**<0.001**
	**Low narcissism vs. Benevolent**	**1.92**	**0.34**	**1.25**	**2.58**	**6.81**	**5.66**	**<0.001**
	**Malevolent vs. Benevolent**	**1.20**	**0.28**	**0.65**	**1.76**	**3.33**	**4.26**	**<0.001**
	**Low narcissism vs. Highly Malevolent**	**0.57**	**0.25**	**0.08**	**1.07**	**1.77**	**2.27**	**0.02**
	**Malevolent vs. highly Malevolent**	**0.89**	**0.25**	**0.39**	**1.38**	**2.43**	**3.52**	**<0.001**
	Malevolent vs. low Narcissism	0.17	0.17	–0.16	0.50	1.19	1.02	0.31
	Age	0.32	0.34	–0.35	0.98	1.37	0.94	0.35
	Gender	–0.40	0.28	–0.95	0.15	0.67	–1.43	0.15
	**Socioeconomic status**	–**0.72**	**0.28**	–**1.27**	–**0.16**	**0.49**	–**2.54**	**0.01**
Highly troubled vs. flourishing	Benevolent vs. low Mach-Psycho	0.10	0.12	–0.14	0.33	1.10	0.82	0.41
	Highly MALEVOLENT vs. Low Mach-Psycho	–0.05	0.17	–**0**.38	0.28	0.95	–0.29	0.78
	Low narcissism vs. Low Mach-Psycho	–0.04	0.02	–0.08	0.01	0.97	–1.58	0.11
	**Malevolent vs. Low Mach-Psycho**	**1.89**	**0.60**	**0.71**	**3.07**	**6.63**	**3.15**	**0.01**
	**Highly malevolent vs. Benevolent**	**2.90**	**0.66**	**1.61**	**4.19**	**18.21**	**4.42**	**<0.001**
	**Low narcissism vs. Benevolent**	**3.40**	**0.65**	**2.12**	**4.67**	**29.83**	**5.23**	**<0.001**
	**Malevolent vs. Benevolent**	**2.42**	**0.62**	**1.20**	**3.64**	**11.23**	**3.89**	**<0.001**
	**Low narcissism vs. Highly Malevolent**	**1.01**	**0.31**	**0.41**	**1.61**	**2.75**	**3.31**	**<0.001**
	**Malevolent vs. Highly Malevolent**	**1.50**	**0.29**	**0.94**	**2.07**	**4.50**	**5.20**	**<0.001**
	**Malevolent vs. Low Narcissism**	**0.53**	**0.22**	**0.10**	**0.96**	**1.70**	**2.39**	**0.02**
	Age	0.49	0.39	–0.27	1.25	1.64	1.27	0.20
	Gender	–0.48	0.34	–1.15	0.18	0.62	–1.43	0.15
	**Socioeconomic status**	–**0.98**	**0.33**	–**1.62**	–**0.34**	**0.38**	–**2.99**	**0.01**

*N = 1,640. ^a^Coded as 1 = male, 2 = female. The flourishing profile was regarded as the reference group. Mach-Psycho, Machiavellianism-Psychopathy. The significant values are highlighted in bold type.*

As shown in Figure 3, students within the low Machiavellianism-psychopathy profile exhibited the highest likelihood of flourishing, and students within the low narcissism profile reported the highest likelihood of being highly troubled. Students within the benevolent and malevolent profiles showed a similar likelihood of being flourishing and troubled, but reported a higher likelihood of flourishing than being vulnerable and highly troubled. For students within the highly malevolent profile, the likelihood of flourishing was surpassed by the likelihood of being troubled, although the likelihood of flourishing was still higher than being highly troubled and vulnerable.

## Discussion

Attempting to develop a comprehensive and person-centered view of the association between the Dark Triad and youth mental health, the current study examined both the Dark Triad profiles and mental health profiles among a large-scale sample of Chinese high school students. This study also examined whether differing Dark Triad profiles may diverge in mental health profiles. Results of the latent profile analyses revealed five Dark Triad profiles and four mental health profiles. Students with differing Dark Triad profiles diverged in mental health profiles.

In terms of RQ1, as expected, we found four mental health profiles: flourishing, vulnerable, troubled, and highly troubled. These profiles partially supported a dual-factor model of mental health, but we did not discover a profile characterized by high scores on both psychopathology and wellbeing (i.e., symptomatic but content). One possible explanation is that emotional restraint and avoidance of extremities are highly valued in Chinese society (20). Under this cultural emphasis, individuals are not expressive and tend to avoid reporting extremely high scores on both negative and positive indicators (66). Moreover, most students belonged to the flourishing profile. This finding indicates that, despite high academic stress and parental expectations, high school students may handle these stressors well and exhibit high academic engagement in the school setting, which is linked to the students’ positive mental health conditions.

In terms of RQ2, rather than the expected three or four profiles, we found five Dark Triad profiles: low Machiavellianism-psychopathy, benevolent, highly malevolent, low narcissism, and malevolent. The features of these profiles partially align with prior research (38) but also exhibit some additional subgroups (e.g., low Machiavellianism-psychopathy and low narcissism). These five profiles may also reflect some unique developmental features of high school students compared to prior research (38, 41, 42), Upon growing older, students may provide more differentiated self-ratings on the Dark Triad (50, 67) given that the traits are associated but distinguishable (31). Moreover, most of the high school students belonged to the benevolent profile. One possible explanation relates to Chinese culture, in which collectivism, cooperative behavior, and harmonious group wellbeing are highly valued (20). In this cultural context, youth are socialized to be “benevolent.” Notably, however, the current findings are possibly influenced by the nature of self-reports, in which students may respond to each item based on socially desirable manners and central tendencies (see “Limitations and Implications” section for elaboration) (68, 69).

Regarding RQ3, partially aligned with the hypothesis, we found that students within the low Machiavellianism-psychopathy profile exhibited the highest likelihood of flourishing, and students within the low narcissism profile reported the lowest likelihood of flourishing. This finding aligns with prior research (31, 36, 57) and indicates that Machiavellianism and psychopathy are both aversive and negative in nature, whereas narcissism shows a distinctive pattern of psychological correlates and is perceived more favorably than Machiavellianism and psychopathy. Moreover, students within benevolent and malevolent profiles showed a similar likelihood of flourishing and troubled profiles. This finding may indicate that, when youth report concurrent high or low levels of the Dark Triad, the levels may not determine the students’ optimal psychological function. After all, youth mental health is a complex issue affected by the interactions of many other personal and contextual factors, such as social support, and that complexity ultimately impacts psychological function (70).

### Limitations and Implications

Several limitations should also be considered when interpreting the results. First, the study overly relies on self-report measures, which may lead to shared method variance and social desirability bias that potentially interfere with the interpretations of Dark Triad profiles and their associations with youth mental health. For instance, prior research has shown that narcissism is positively associated with social desirability, whereas Machiavellianism and psychopathy are negatively correlated with social desirability (68). These findings may explain why the current study reveals several insignificant differences in narcissism across the Dark Triad profiles, whereas Machiavellianism and psychopathy do not. In a similar vein, the research findings gathered should be carefully interpreted within cultural boundaries. Extensive existing documentation already establishes that culture plays a profound influence in shaping the self and personality construct (27, 71). Accordingly, collectivistic societies, such as China, heavily emphasize the group over the self, so youth may report relatively fewer variances in narcissism compared to youth from individualistic societies (27, 57). Second, this study is based on a cross-sectional design, and the longitudinal association between the Dark Triad and youth mental health remains unknown. Based on a cross-sectional analysis, conclusions regarding the directions of these associations therefore cannot be inferred. Prior research has suggested that dark personality characteristics are the consequence of changes in antisocial behavior (72), but future studies should broaden this line of research by leveraging a longitudinal design to clarify the directions of the Dark Triad and youth mental health (incorporating both positive and negative outcomes). Finally, the current study focuses on exploring individual differences and their associations with youth mental health, but the underlying pathways and conditional processes of the associations are underexplored. Future studies should broaden the current study by, for instance, examining the contexts in which these developments occur, such as in parent-child relationships, teacher-student relationships, and peer interactions (73). Investigating these contexts is vital to understanding mechanisms and conditional processes toward healthy adolescent development and can help identify possible targets for intervention and prevention programs.

Despite these limitations, the current study conveys essential theoretical and practical implications. First, some researchers, such as Muris et al. (30), have questioned the structure of the Dark Triad since narcissism is far less antisocial in nature than Machiavellianism and psychopathy (30, 74). Studying the Dark Triad in a person-centered approach conceptually contributes to the discussion regarding the exclusion of narcissism from the Dark Triad. Further, most existing studies have investigated the associations between the Dark Triad and psychological maladjustment (75), but little research has focused on the association of the Dark Triad with positive psychological outcomes. The current study therefore aimed to capitalize on the dual-factor model of mental health to extend prior research by gaining a more conceptually rich understanding of the Dark Triad with both positive and negative psychological outcomes. Practically, exploring the Dark Triad profiles based on their natural combinations can unmask group-specific associations between malevolent personality traits and youth mental health—associations otherwise often obscured by latent entities and global scores subsumed under specific facets. For instance, by measuring the presence of the Dark Triad among students, practitioners and health professionals could precisely locate “highly troubled” adolescents and keep them on track for early identification and subsequent mental health intervention.

## Conclusion

Using a dual person-centered approach, this study exhibits a comprehensive view on the association between high school students’ mental health and the Dark Triad. Specifically, students with low Machiavellianism and psychopathy report better mental health than students with other profiles, whereas those with low narcissism exhibit a high possibility of being highly troubled.

## Data Availability Statement

The raw data supporting the conclusions of this article will be made available by the authors, without undue reservation.

## Ethics Statement

The studies involving human participants were reviewed and approved by the Wenzhou University of Technology. Written informed consent to participate in this study was provided by the participants’ legal guardian/next of kin.

## Author Contributions

YH conceived and drafted the manuscript. XL performed the statistical analyses and critically revised the manuscript. Both authors read and approved the final draft of the manuscript.

## Conflict of Interest

The authors declare that the research was conducted in the absence of any commercial or financial relationships that could be construed as a potential conflict of interest.

## Publisher’s Note

All claims expressed in this article are solely those of the authors and do not necessarily represent those of their affiliated organizations, or those of the publisher, the editors and the reviewers. Any product that may be evaluated in this article, or claim that may be made by its manufacturer, is not guaranteed or endorsed by the publisher.

## Footnotes

1 The dark personality literature is still swiftly evolving. For instance, investigations on the Dark Tetrad and Light Triad (faith in humanity, humanism, and Kantianism) have gained increasing popularity in the scientific community (32, 76). In the current study, we aimed to explore the Dark Triad profiles in a sample of Chinese high school students as an extension of the research findings from other populations (38), and as a confirmatory investigation to respond to the inconsistent findings in this field (42, 43). Focusing on the Dark Tetrad and Light Triad, albeit interesting, ultimately extends beyond the current focus. Moreover, validating the targeted measurements in Chinese youth remains scarce, making this initiative less practical. Recent studies have, however, revealed a unique explained variance of sadism over the Dark Triad (77) and demonstrated meaningful Dark Tetrad profiles (41). Accordingly, future investigations on a comprehensive and person-centered view of the association of the Dark Tetrad profiles (may also be extended to the Light Triad) with youth mental health are warranted.
